# Cultural variations in global and local attention and eye-movement patterns during the perception of complex visual scenes: Comparison of Czech and Taiwanese university students

**DOI:** 10.1371/journal.pone.0242501

**Published:** 2020-11-16

**Authors:** Jiří Čeněk, Jie-Li Tsai, Čeněk Šašinka

**Affiliations:** 1 Department of Social Studies, Faculty of Regional Development and International Studies, Mendel University in Brno, Brno, Czech Republic; 2 Laboratory of Eye-Movements and Reading, Centre for the Mind, Brain and Learning, Department of Psychology, National Chengchi University, Taipei, Taiwan (R.O.C.); 3 Division of Information and Library Studies, Faculty of Arts, Masaryk University, Brno, Czech Republic; University of New Brunswick Fredericton, CANADA

## Abstract

Previous research on cross-cultural differences in visual attention has been inconclusive. Some studies have suggested the existence of systematic differences in global and local attention and context sensitivity, while others have produced negative or mixed results. The objective in this study was to examine the similarities and differences in holistic and analytic cognitive styles in a sample of Czech and Taiwanese university students. Two cognitive tasks were conducted: a Compound Figures Test and a free-viewing scene perception task which manipulated several focal objects and measured eye-movement patterns. An analysis of the reaction times in the Compound Figures Test showed no clear differences between either sample. An analysis of eye-movement metrics showed certain differences between the samples. While Czechs tended to focus relatively more on the focal objects measured by the number of fixations, the Taiwanese subjects spent more time fixating on the background. The results were consistent for scenes with one or two focal objects. The results of a correlation analysis of both tasks showed that they were unrelated. These results showed certain differences between the samples in visual perception but were not as systematic as the theory of holistic and analytic cognitive styles would suggest. An alternative model of cross-cultural differences in cognition and perception is discussed.

## Introduction

Multiple research findings (for review see [1, 2]) suggest the existence of systematic cross-cultural differences in cognitive processing around the world. Much of the research investigates the cultural differences between “the East” (i.e. China, Japan, South Korea) and “the West” (i.e. Canada, USA, Western Europe) and anticipates the existence of systematic and relatively stable differences in cognition or cognitive styles.

It is uncertain which factors cause variations in cognitive processes. It is reasonable to assume that they are based on the interplay of multiple factors that include sociocultural, environmental and biological influences, such as philosophical tradition [1], parent-child interaction [3, 4], socioeconomic status and personal wealth [5], literacy [6], the complexity of the physical environment [7], differences in the anatomical and functional aspects of the central nervous system [8, 9], or means of subsistence [10, 11]. Probably the most used explanatory factors for the existence of cultural differences in cognition are the cultural syndromes of individualism and collectivism (independent and interdependent self, respectively) [12, 13]. The theory of individualism/collectivism (and independence/interdependence, respectively) suggests fundamental differences in how individuals relate to society, how this relationship is constructed, and whether individuals or groups are the basic units of analysis. Collectivistic cultures are characteristic for emphasizing interdependence and orientation in social groups (e.g. extended families, communities). In individualistic cultures, emphasis is placed on individual independence and autonomy [12, 14].

Despite the growing body of literature on the topic, research in this area is far from complete. Besides the uncertain causal relationships between cognitive and cultural and environmental variables, at least two other “weak spots” or points of interest can be found in the existing body of research on holistic and analytic cognitive styles. First, despite several exceptions [5, 15–21], the research almost exclusively focuses on a simplified and reductionist comparison of “Western” (North America and Western Europe) and “Eastern” (East and Southeast Asia) populations, thereby ignoring all other cultural regions and the possible variations in cognitive processes in these regions. Furthermore, some evidence exists of the presence of differences in perceptual and cognitive processes, not only between people from different countries but also different regions within these countries [10, 19, 22]. Second, several recent studies have shown contradictory or mixed results, or minor effect sizes [21, 23–27], and call the theory of cognitive styles into question. Some advocate the prevalence of universal, bottom-up processes, while others acknowledge the need for replication in research on cognitive styles.

At least two models have attempted to explain the differences in cognitive style. Nisbett [1, 2] formulated a model (in the present paper, referred to as the “general holistic–analytic model”) of cognitive styles that is based on a vast amount of empirical evidence and that postulates systematic differences exist in cognitive processes between Easterners (holistic cognitive style) and Westerners (analytic cognitive style). More specifically, it describes differences in: (a) object categorization [28, 29], (b) reasoning about contradictions [30], (c) field dependence and object-background differentiation [31], (d) context sensitivity and selective attention on objects and relationships, (e) processing of global and local attributes of objects [32], (f) change detection [33, 34] and (g) memory [35]. If we accept the axioms of the general holistic–analytic model, holistic individuals (compared to their analytic counterparts) should: (a) use more intuitive and less rule-based strategies in object categorization, (b) use dialectical thinking instead of rules of formal logic, (c) have more problems with separating objects from the background, (d) focus more on the background and relationships between objects and less on the salient (or focal) objects and their attributes [15, 36], (e) focus more on the global and less on the local features of objects, (f) be more sensitive to the contextual and less to the focal object changes, and (g) recall objects in complex scenes less successfully. An important attribute of this model is that these differences should be coherent. This means that if a holistic individual focuses relatively more on the global features, he or she should also focus relatively more on the background. In other words, the scores obtained by multiple methods should be (cor)related.

Kozhevnikov et al. [37] proposed an alternative model (in the present paper, referred to as the “hierarchical–ecological model”) of cognition by emphasizing the ecological nature of cognitive style, viewing cognitive styles as patterns of adaptation to the environment. According to this view, cognitive style is environmentally dependent, flexible and task specific. This model is based on Nosal’s [38] earlier model; she proposed a hierarchical model of cognitive styles: a cognitive–style matrix that organizes cognitive styles along two axes or levels consisting of information processing (perception, concept formation, higher-order processing, metacognitive processing) and cognitive style families (context dependence and independence, rule-based and intuitive processing, internal and external locus, integration and compartmentalization). The most used cognitive styles are positioned along these axes. According to Kozhevnikov’s model, the different cognitive styles would not necessarily have to (cor)relate since an environment might, for example, support both the development of global processing (holistic characteristic) and focus on salient objects (analytic characteristic).

In this research, the possible cross-cultural variations in two cognitive processes, (a) global and local attention and (b) context sensitivity, were examined in samples of Czech and Taiwanese university students. According to the research conducted by Hofstede [39], the Czech Republic is relatively high in individualism (individualism score = 58), while Taiwan is a typically collectivistic country (individualism score = 17). The selected samples therefore reflect the above-mentioned need to investigate samples beyond the traditional “USA vs. China/Japan” borders, which is also logical from a theoretical point of view. Even though both countries have experienced unique and sometimes turbulent periods in recent history (wars, waves of migration, communist dictatorship, etc.) and are not seen as typical representatives of individualistic or collectivistic cultures, we might still assume the presence of differences in cognitive style. The psychology of people in the Czech Republic has been shaped by typically European influences, such as Christianity and Greek philosophy [1]. The country has a relatively less complex physical environment [7] and is a typical wheat culture [11]. Taiwan, however, is still part of the Asian cultural space, with Buddhism and Taoism as the main religions, a tradition of Chinese philosophy, a relatively more complex environment, and rice as a main means of subsistence. As such, we might expect Czechs (individualistic country) to perceive more analytically, while the Taiwanese (collectivistic country) to perceive more holistically.

To investigate global and local attention, a hierarchical Navon figures test was used. In the present study, we refer to our version of the PC-administered Navon figures test as the Compound Figures Test (CFT; see details in Materials and Methods section). This test presents figures at two hierarchical levels: global and local [32, 43]. The global level is generally represented by a letter (e.g. “H”), number (e.g. “3”), or shape (e.g. square). The global-level feature of the figure comprises multiple local-level features of the same type (e.g. local letters which form a global letter, or local numbers which form a global number).

Tests using hierarchical figures have been previously used in multiple cross-cultural examinations focused on processing the global and local features of objects [32, 40, 41]. With some exceptions [24], they report a relative advantage in the processing speed of global characteristics of stimuli (global advantage/precedence/preference) in Asian subjects compared to Westerners. The cross-cultural differences in context sensitivity (attention to an object vs. attention to the background) were examined using natural scenes (free-viewing paradigm) combined with eye-movement recording in a design similar to previous research conducted by other authors [15, 23, 36]. Some of the research found distinct differences in the eye-movement patterns between Chinese and Americans [36] and Chinese and Africans [15], while other enquiries supported a contradictory hypothesis on the lack of any systematic cultural differences in scene viewing [23].

The formulation of hypotheses in the present paper is based upon the general holistic–analytic model [2]. We formulate the hypotheses according to this model and not the competing model by Kozhevnikov [37] because most cross-cultural studies on the topic are also based on this model and it offers a strong empirical basis for the formulation of such studies. As mentioned above, we applied two methods to assess the cognitive style of respondents and expected that performance in these methods would be modulated by cultural group. The hypotheses were formulated with respect to the metrics (scores) obtained by these methods. To examine the global vs local attention, we applied a CFT that has two main metrics to work with: a global precedence score (calculated as a difference in global and local reaction times; see the Stimuli section for details) and an error rate. In the second method, we investigated context sensitivity (attention to an object vs. attention to the background) in free-viewing task with a set of complex natural scenes (composed of one or two objects and a background; see the Stimuli section for details) combined with eye-movement measurement. The measurements included several common eye-tracking metrics, namely the number of first fixations, number of fixations, fixation time and transitions between parts of the scenes.

Global vs local attention (CFT)–a) The Taiwanese respondents should demonstrate a stronger global preference than Czech participants in CFT processing speed [32]. CFT–b) No significant differences in the error rate of responses between the two groups were expected [32]. Context sensitivity (scene perception)–The Taiwanese respondents should: a) make fewer first fixations on a focal object (percentage of first fixations on a focal object), b) make fewer focal object fixations (average number of focal object fixations), c) fixate focal objects for a shorter time (average focal object fixation time), d) make more background fixations (average number of background fixations), e) fixate backgrounds for a longer time (average background fixation time), and f) make more focal object to background transitions. In the case of stimuli with two focal objects, the Taiwanese were expected to h) make more direct transitions between both focal objects. In addition, because a cognitive style is defined according to the general holistic–analytic model as a complex set of behaviours, g) a correlation was expected between the eye-movement measurements in the perception of scenes and the global preference score of the CFT.

Scene perception related hypotheses a–e) were formulated according to the research by Chua et al. [36] and Duan et al. [15]. Hypotheses f–h) were based on the general holistic-analytic model, but were not, to our best knowledge, previously tested [1, 2, 42]. They reflect the assumption that holistic cultures “*tend to engage in context-dependent and holistic perceptual processes by attending to the relationship between the object and the context in which the object is located*” [42, p.1]. Furthermore, if the holistic and analytic cognitive style, as defined by the general holistic–analytic model [1], represents the quality of cognitive processes, where holistic perceivers compared to their analytic counterparts should, for example, perceive the global characteristics of stimuli relatively more quickly and also focus more on the relationships between objects in a scene, then the scores obtained by the methods measuring these qualities should also correlate with each other (hypothesis g)). If this is not the case, the hierarchical–ecological model [37] of cross-cultural differences in cognition might be more plausible.

Our research contributes by improving the understanding of cultural similarities and differences in visual attention and perception in at least three ways: (i) it is one of relatively few studies that explores multiple facets of cognitive style [24], (ii) it is, to our best knowledge, the first study to compare Asians and Central Europeans by measuring eye-movement patterns in viewing a scene, (iii) it is the first cross-cultural eye-tracking research that controls the number of focal objects in a scene (1 or 2).

## Materials and methods

The Research Ethics Committee of Masaryk University has reviewed the application to conduct the research project and has approved this project (Proposal No.: 0257/2018) to be conducted on 13 March 2019. Informed consent was obtained in writing from all participants.

### Stimuli

Besides the two experimental tasks described in this section, a personal questionnaire was administered and asked respondents to state their gender, age, experience in living in a foreign country (more than one year, yes or no), the size of their household before entering university and the current size of their household. All tasks were administered in either Czech or traditional Chinese according to the cultural background of the participant.

#### Compound Figures Test

To assess global and local distribution of attention, we applied a Compound Figures Test: a numerical PC version of the original Navon test [43]. CFT has been applied in previous studies [44–47]. The test was administered using the Hypothesis software [48, 49].

**Fig 1:**
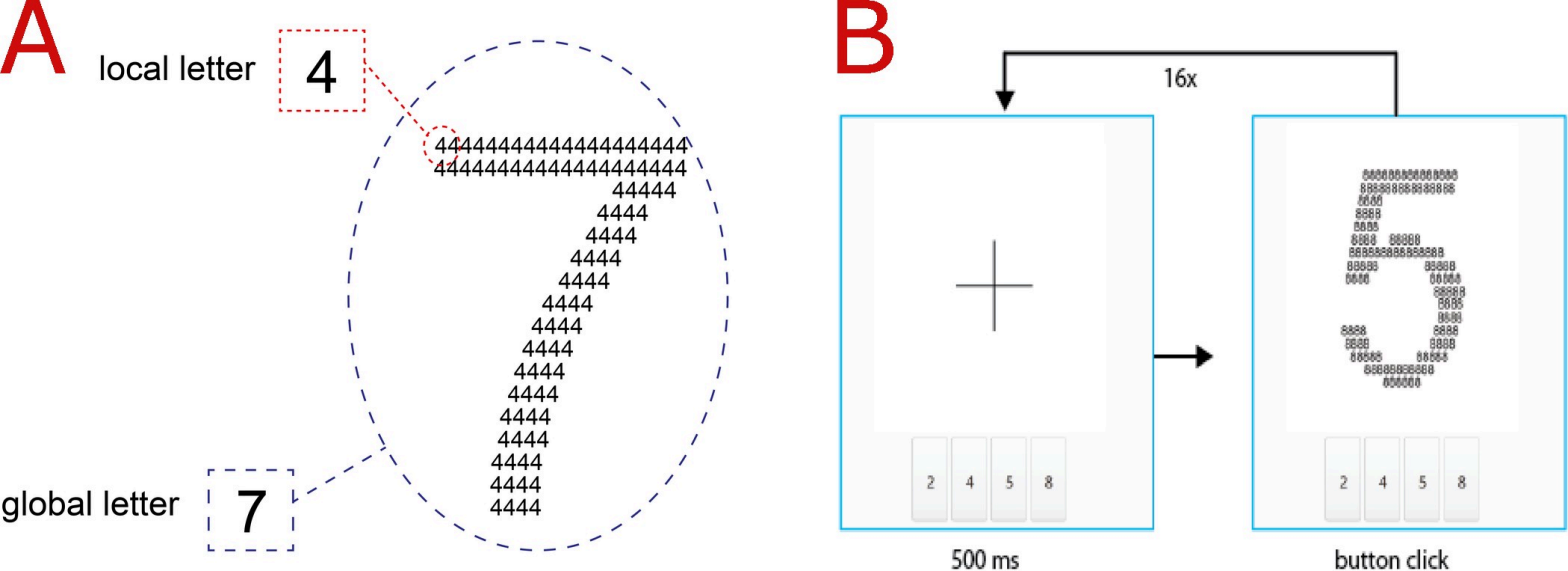
CFT stimulus example and procedure. (A) CFT stimulus. (B) CFT procedure. A fixation cross is displayed for 500 ms before each stimulus. After the fixation cross is displayed, a compound letter is presented. Depending on the task (local vs. global), the participant identifies the local or global feature of the stimulus and responds by pressing the corresponding number.

In each task, the participant was presented with a large (global) number (Fig 1A) comprising multiple smaller (local) numbers. In the CFT, only four numbers were used as global and local numbers: 2, 4, 5 and 8. The participant was instructed to identify either the global (global task) or local level of the stimulus (local task) and select from four possible responses (one correct answer and three distractors) the correct answer as quickly as possible with a mouse-click. Before the test, the participant was given three practice trials and received feedback whether their response was correct. The participant did not proceed unless he or she selected the correct answer in each practice trial. The entire test comprised six practice tasks (three local and three global) and 32 test trials (16 for local and 16 for global processing). The local task preceded the global task in all cases. A fixation cross was displayed for 0.5 seconds before each trial (Fig 1B).

The reaction times and error rate of the responses were measured in each test. The mean speed and error rate of the local and global tasks were calculated separately. Four average values were therefore recorded for each participant: global reaction time (RT), local reaction time, global error rate, and local error rate. The main score, or the global preference score, was calculated in the CFT [24, 32] as local RT-global RT and served as a major indicator of local and global attention. Let us remind that we assumed the Taiwanese respondents would demonstrate a stronger global preference than Czech participants in CFT processing speed. The error rate of responses was a control variable, i.e. a high number of mistakes indicated the decreased validity of the test results due to, for example, less motivation or misinterpretation of the test. No significant differences in the error rate of responses between the two groups were therefore expected.

#### Complex scenes

**Fig 2:**
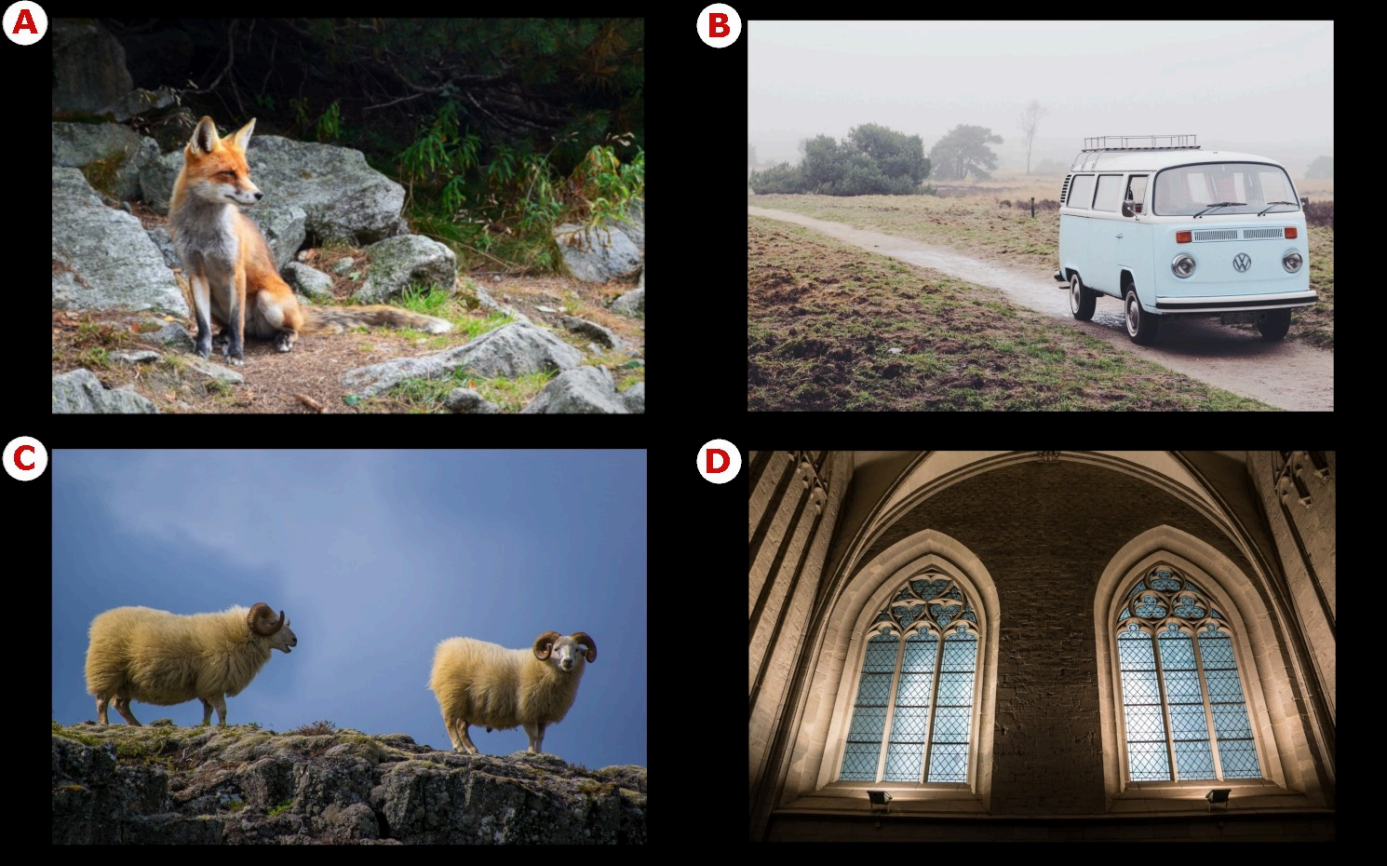
Examples of real-world scenes. (A), (B) Samples of one focal object scenes. (C), (D) Samples of two focal object scenes. Copyright statement: All images used in this figure are free for commercial and personal use.

To investigate the possible cultural differences in context sensitivity of the two samples, 60 images of real-world scenes were used. The pictures were downloaded from free online image depositories (wallpaperflare.com, pxhere.com). Half of the scenes were similar to those used in other research [15, 23, 36], consisting of one focal object (animals or inanimate objects such as vehicles, buildings, doors and windows on a facade) against backgrounds of different complexity (Fig 2A and 2B) from relatively uniform to moderately complex. The other half of the stimuli were similar but contained two focal objects (Fig 2C and 2D) of the same category. In the scenes containing one focal object, the object was positioned either centrally (10 images), at the left (9 images), or at the right (11 images). In the scenes with two focal objects, the positions of the objects were not controlled. The scenes for both groups were the same size (1024 x 688 px) and the scenes were placed on the black background.

The participants were instructed to view a series of pictures and evaluate how much they liked each picture on a scale of 1 to 5 (1 –very good, 5 –very poor). These data were not analysed. Two practice runs preceded the testing. A fixation cross was displayed before each test until the moment the participant fixated on it. After the participant successfully fixated on the cross, it disappeared, and an image was displayed for four seconds.

This timing was selected according to previous research on perceiving scenes using different durations to display stimuli. For example, Chua et al. [36] and Evans et al. [23] displayed the stimuli for three seconds, and Duan et al. [15] displayed stimuli for five seconds. Chua et al. [36] showed that the proportion of object fixations varied throughout the course of testing. After a stimulus was displayed, participants mostly fixated on focal objects (bottom-up process driven by salience) for around 300–400 ms. The proportion then varied throughout the testing, and any potential differences in fixation count and duration may have disappeared after a long enough (e.g. 10 s) exposure to the scene [26].

The testing was presented in two separate batches: pictures with one focal object and pictures with two focal objects. A one-minute break was given between the batches. The sequence of batches was balanced: half of the participants first viewed the batch with one focal object and the other half viewed the batch with two focal objects. The sequence of pictures in each batch was pseudo-random. The eye-movement data were recorded for each test.

### Apparatus

The CFT data were collected using Hypothesis software (see above) and the Google Chrome web browser. The participants viewed the stimuli without a chinrest. In Taiwan, a 19″ (EIZO FlexScan S1901) LCD monitor with a resolution of 1280 x 1024 was used. The viewing distance was approximately 65 cm. In the Czech Republic, a 22″ (AOC I2267FW) monitor with a resolution of 1680 x 1050 was used. The viewing distance was approximately 70 cm. The size of the instructions with illustrative examples and size of the stimuli (the compound hierarchical letters) were the same for both groups (900 x 675 px and 440 x 500, respectively).

In Taiwan, eye-movements were tracked with an EyeLink 1000 desktop type eye-tracker. The stimuli were presented on a 19″ (ViewSonic P95f+) CRT monitor with a resolution of 1024 x 768. In the Czech Republic, eye-movements were tracked using an SMI Red eye-tracking system with an integrated 22″ monitor (Dell P2213) with resolution of 1680 x 1050 px. The size of stimuli was same in both countries (1024 x 688 px). A chin rest positioned approximately 70 cm away from the monitor was used to minimize any disruptions caused by head movements. The visual angle of stimuli in Czech Republic was approximately 31.5° horizontally and 21.5° vertically. The visual angle of stimuli in Taiwan was approximately 30.1° horizontally and 21.8° vertically. In both countries, the sampling rate was set to 500 Hz, with 9 points of calibration. The minimum accuracy of calibration was set to 1° of visual angle. The same threshold was used for all participants.

### Participants and procedure

The test battery was translated using the parallel translation method, which is commonly used to reduce method bias in cross-cultural test adaptations [50, 51]. Two bilinguals translated the test materials (test instructions). Both versions were then assessed for any potential differences. If the translations differed, the differences were discussed by the research team until a consensus on optimal translation was reached.

The research participants in both countries were recruited through university groups on social networks. Participation was limited to people of Czech or Taiwanese nationality possessing no eye-diseases or colour blindness and normal or fully corrected vision. A formal administration procedure was created, and the process of administration in both countries, including the instructions given to participants and the task sequence, remained the same. The test battery was administered in both countries by a local administrator (Czech and Taiwanese, respectively) to prevent a potential method bias [52]. Administrators of the test battery at both sites were also trained to administer the battery in the same manner. The test battery was administered in the following sequence: after entering the laboratory, participants a) read and signed an informed consent form, b) filled in a sociodemographic questionnaire, c) completed the CFT, and d) completed the complex scenes task.

The minimum required sample size was estimated before the experiment commenced using G*Power 3.1 [53] for ANOVA, fixed effects with effect size = 0.25, α = 0.05, Power = 0.8, and 4 groups (2 [area: object vs. background] ˟ 2 [nationality]). The required total sample size was 128 respondents. In total, we gathered data from 129 participants (60 Taiwanese, 69 Czechs). The detailed procedure of data cleaning and number of participants in each of the statistical analyses are described in the respective sections of the Results. See Table 1 for a summary of the sample’s characteristics.

**Table 1:** Research sample characteristics summary.

Variable	Taiwanese (N = 60)	Czech (N = 69)
% of women	71.6	71.0
Age–Mean (SD)	21.1 (2.07)	21.5 (2.65)
% of participants living abroad for longer than a year	8.3	14.5
Household size–Mean (SD)	4.1 (1.16)	4.0 (1.16)

In the present paper, we used the following statistical programmes: G*Power 3.1 [53] for power analysis; R 3.5.2 [54] for eye-tracking data pre-processing and statistical analysis; Ogama 5.0.1 [55] for eye-tracking data cleaning, ROI (Regions of Interest) definition and fixation calculation. Stimuli, data files, R scripts and procedural descriptions are publicly available in the OSF repository (https://osf.io/eubwn/; DOI 10.17605/OSF.IO/EUBWN).

We applied the following analyses. The cultural differences in the CFT were tested with independent samples and paired sample t-tests. Cultural differences in the scene perception task were tested using mixed ANOVAs with one between-subject factor (cultural group) and one within-subject factor (ROI), post-hoc tests, and independent sample t-tests. In all analyses, partial eta squared (ηp^2^; ANOVA) and Hedges’ g (g; post hoc tests, t-tests) effect sizes were calculated. Finally, for an exploratory analysis of relationships between the main eye-tracking metrics and other variables, we used linear regression.

## Results

### Analysis 1: Compound Figures Test

In the first stage, the average error rate and average reaction time (speed) scores were computed for the local and global tasks (16 trials for each subtest). The error rate score was taken as an indication that the participant understood the task correctly. If the error rate of a certain participant was high, the participant was excluded from further analysis. Before the data were cleaned, the overall average error rate was 0.9% for the local task and 3.0% for the global task. The Taiwanese participants had a slightly higher average error rate in both local (1.0%) and global (4.9%) tasks compared to the Czech participants (0.8% for local and 2.2% for global). The maximum number of errors in the local task was one (corresponding to an error rate of 6.25%–out of 16 trials in total). Several participants (6 Taiwanese, 2 Czechs) had higher error rates in the global task. The number of errors in a task greater than four (corresponding to a 31.3% error rate–out of 16 trials in total–or higher) cannot be attributed to a temporary lapse in attention or “mouse misclick”, but rather suggest a misunderstanding in the nature of the task. When we removed these eight participants from the data set, the overall average error rate in the global task dropped to 3.4%. The average error rate of Taiwanese (1.2% for local and 1.5% for global) and Czech (0.8% for local and 1.3% for global) participants was almost identical, and the differences were not significant, with negligible effect sizes (global task: t(101.59) = 0.26, p = 0.795, g = 0.048; local task: t(99.11) = 0.69, p = 0.487, g = 0.130). In the next stage of CFT data cleaning, we examined the average processing speed of global and local tasks. One Taiwanese participant was excluded from further analysis as an extreme outlier (reaction time for a local task more than 11 standard deviations from the group mean).

After data cleaning, the data from 120 respondents (53 Taiwanese, 67 Czechs) were analysed according to reaction time. The data for reaction times are summarized in Table 2. The data shows that both the reaction times and variability were generally higher in the Taiwanese sample. The mean reaction times were also higher in the local task. To test the differences in global vs. local RTs in each group, we performed two paired t-tests separately for both nationalities. The differences were significant for global vs local RTs in both the Czech sample (t(66) = 8.98, p = 4.692e-13),13 with a large effect size (g = 0.936), and for the Taiwanese sample (t(52) = 6.89, p = 7.413e-9),9 with a medium effect size (g = 0.699).

**Table 2:** Summary of statistics for reaction times and global preference by nation (in seconds).

Measurement		Taiwanese (N = 53)	Czech (N = 67)
Local RT (sec)	Mean	2.06	1.02
	SD	0.255	0.132
	Median	2.04	0.97
	IQR	0.242	0.143
Global RT (sec)	Mean	1.89	0.89
	SD	0.229	0.139
	Median	1.86	0.87
	IQR	0.324	0.210
Global preference	Mean	0.17	0.13
*(Local RT–Global RT)*	SD	0.180	0.116
	Median	0.16	0.13
	IQR	0.252	0.143

For reaction times, we subsequently calculated the global preference score using the *local RT*-*global RT* equation, a procedure used in multiple studies with Navon-type hierarchical stimuli [24, 32, 56, 57]. We applied an independent t-test to determine the differences in global processing between both groups. No significant differences were found between the Taiwanese (M = 0.17, SD = 0.180) and Czech (M = 0.13, SD = 0.116) participants, t(84.646) = 1.51, p = 0.136. The effect size was small (g = 0.289).

### Analysis 2: Complex scenes

#### Eye-movement data pre-processing

**Fig 3:**
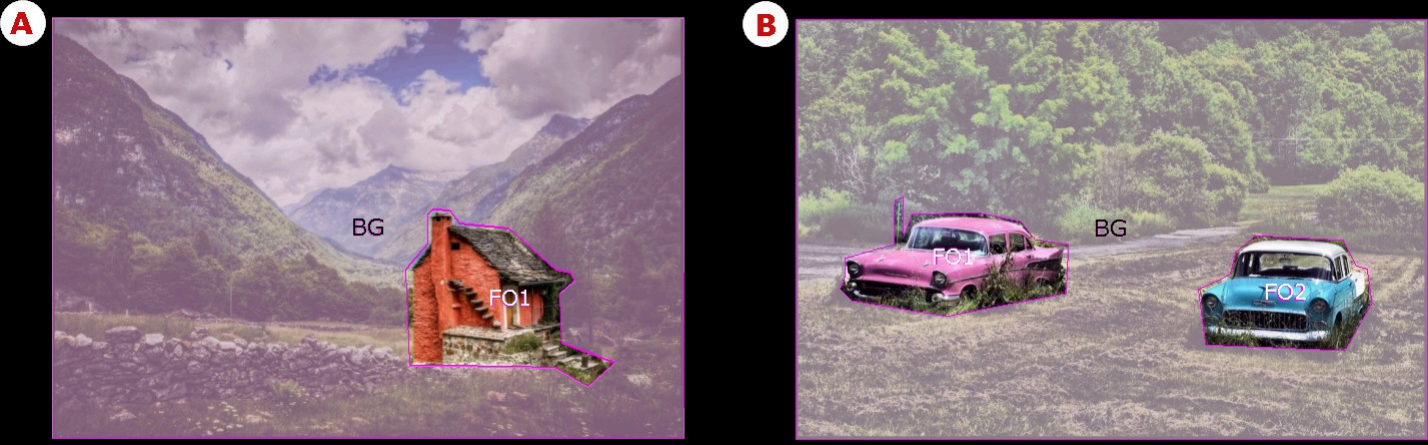
ROIs. (A) Sample one focal object image with ROI. (B) Sample two focal objects image with ROI. Copyright statement: All images used in this figure are free for commercial and personal use.

The differences in data format created by using two different eye-tracking systems forced us to pre-process the eye-movement data before calculating the eye-metrics in Ogama. For this purpose, R 3.5.2 [54] was used. The cleaned data was subsequently imported into Ogama [55]. In Ogama, the following steps were performed: a) data loss analysis, b) definition of ROIs, c) calculation of fixations, d) fixation detection verification. Data loss in the entire sample was relatively low. In the case of Czech participants, data loss varied between 0.13 and 13.77% (mean = 2.89), and in the Taiwanese participants, between 0.02 and 6.99% (mean = 2.91). Two (stimuli with one focal object) or three (two focal objects) ROIs were defined. The ROIs for focal objects were defined around their contours, and the background ROI covered the entire image except for the focal objects and black borders (Fig 3).

Fixations were calculated next. Ogama uses a dispersion-type algorithm [58] to detect fixations. We used the settings suggested by Popelka [59]: maximum distance of 15 pixels, minimum number of 40 samples, size of 31 pixels for the fixation detection ring, automated elimination of first fixation and no merging of consecutive fixations. The number of fixations was checked for each participant and stimulus to identify participants with potential problems in fixation detection (extremely low or high numbers of fixations). Nine participants (8 Czech, 1 Taiwanese) were discarded from further analysis.

As mentioned above, the data for this task were cleaned using a two-step procedure. We first conducted a data loss analysis and then calculated fixations, verifying whether they were correct. From this, we eliminated 13 participants because of the quality of their eye-tracking data. The most common reason for excluding participants was problematic detection of fixations: 8 participants indicated an extremely low number of fixations per trial (e.g. 0 or 1), while the common numbers of fixations per trial were much higher (entire sample median = 12). We excluded participants with median fixations per trial of less than 9. One respondent demonstrated the opposite behaviour: an extremely high number of fixations per trial (median = 22). Both effects were clear indications of a problem in detecting fixations (caused, for example, by shimmering glasses). We also lost the data of three participants due to system error during recording. One participant was discarded for high data loss (13.8% of lost data). After the participants with faulty and missing eye-tracking data were removed, the final analysed sample comprised 116 participants (58 in both groups).

#### Eye-movement data analysis

We expected that Taiwanese and Czechs would show different eye-movement patterns, suggesting differences in visual attention between both groups. More specifically, we analysed the percentage of first fixations on focal objects, the numbers of fixations on focal objects and backgrounds, the focal object and background fixation time, and the transitions between the ROIs. Because two different types of stimuli based on number of focal objects were used, stimuli with one or two focal objects were analysed separately. The parameters for all eye-tracking metrics and for both types of stimuli are summarized in Table 3.

**Table 3:** Summary statistics of eye-tracking metrics for all stimuli according to nationality (fixation time in milliseconds).

Stimulus type		One focal object Mean (SD)		Two focal objects Mean (SD)	
Nationality		Czech	Taiwanese	Czech	Taiwanese
Fixations	% of first fixations on FO	91.0 (8.2)	92.6 (7.2)	98.7 (3.9)	98.3 (2.9)
	Number of FO fixations	8.2 (1.3)	6.8 (1.1)	8.7 (1.7)	7.5 (1.1)
	Number of BG fixations	4.6 (1.2)	4.6 (1.3)	4.0 (1.1)	4.5 (1.1)
	FO fixation time	1949 (383)	2023 (353)	1982 (436)	2035 (299)
	BG fixation time	966 (264)	1218 (290)	838 (235)	1138 (231)
Saccades	Number of within FO saccades	6.3 (1.3)	4.5 (1.0)	4.4 (1.1)	3.2 (0.8)
	Number of within BG saccades	2.8 (1.0)	2.6 (1.2)	2.2 (0.9)	2.3 (0.9)
	Number of FO–BG transitions	3.7 (0.7)	3.6 (0.7)	3.7 (0.8)	3.9 (0.7)
	Number of FO–FO transitions	NA	NA	2.4 (0.8)	2.0 (0.6)

#### One focal object

We first calculated the proportions of first fixations from all first fixations on the focal object. The data shows that in most cases, both Czech (M = 91.0, SD = 8.2) and Taiwanese (M = 92.6, SD = 7.2) participants first fixated on the focal objects. The differences were not significant: t(114) = 1.163, p = 0.247, with a small effect size (g = 0.214). The focal object in most cases was first fixated on by both cultural groups.

**Fig 4:**
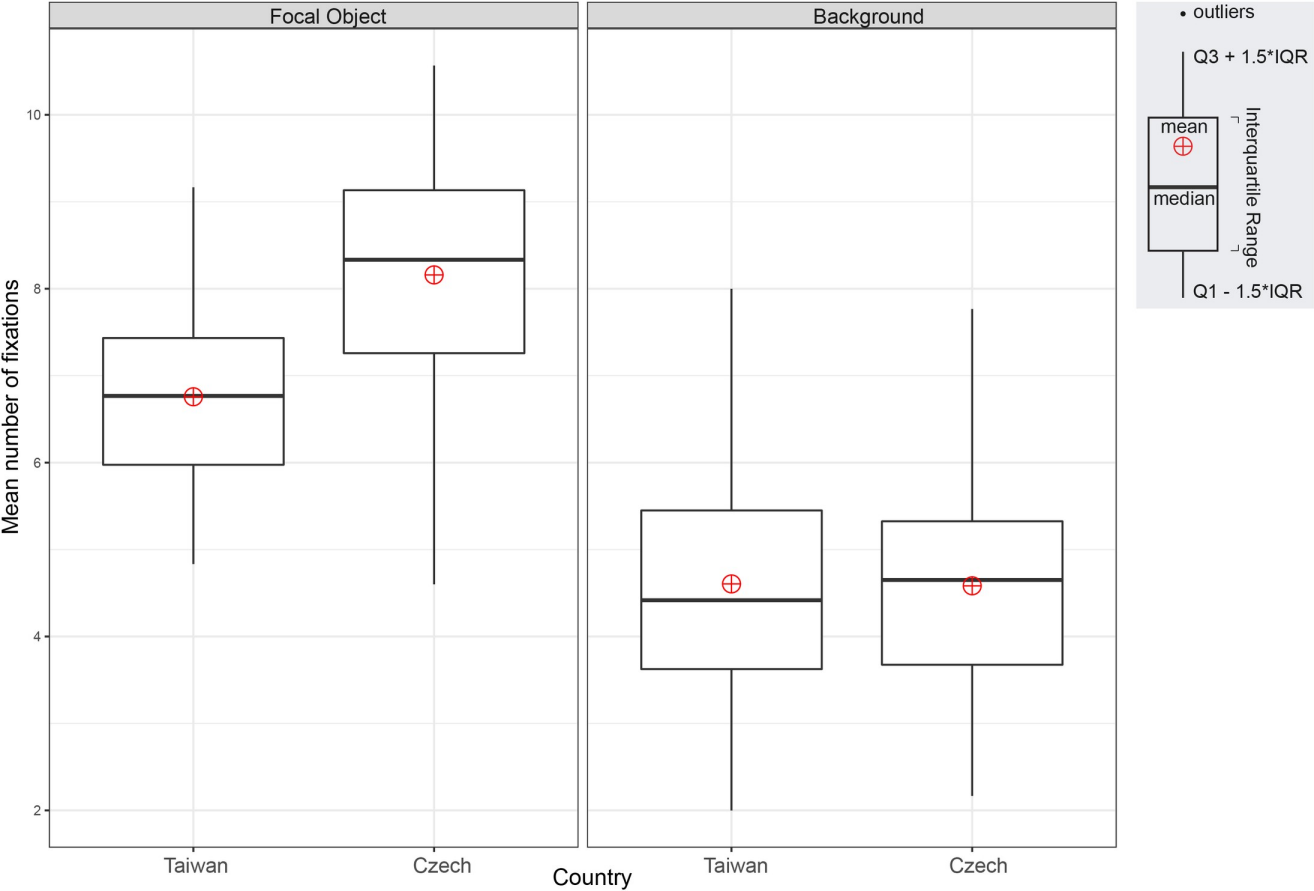
Mean number of fixations: One focal object stimuli.

Both groups showed significantly more fixation counts on focal objects than the backgrounds in the mean number of fixations: F(1, 228) = 308.78, p = 2e-1616, ηp^2^ = 0.58. The main effect of culture was significant: F(1, 228) = 17.92, p = 3.34e-0505, ηp^2^ = 0.07, as was the interaction between culture and ROI: F(1, 228) = 19.07, p = 1.91e-0505, ηp^2^ = 0.08. Fig 4 indicates that the Czech participants (M = 8.2, SD = 1.3) made significantly more focal object fixations than the Taiwanese (M = 6.8, SD = 1.1), t(114) = -6.229, p = 8.079e-0909, with a large effect size g = 1.15. No significant differences were found between the Czech (M = 4.6, SD = 1.2) and Taiwanese (M = 4.6, SD = 1.3) participants in the number of background fixations, t(114) = -0.093, p = 0.93, g = 0.02.

**Fig 5:**
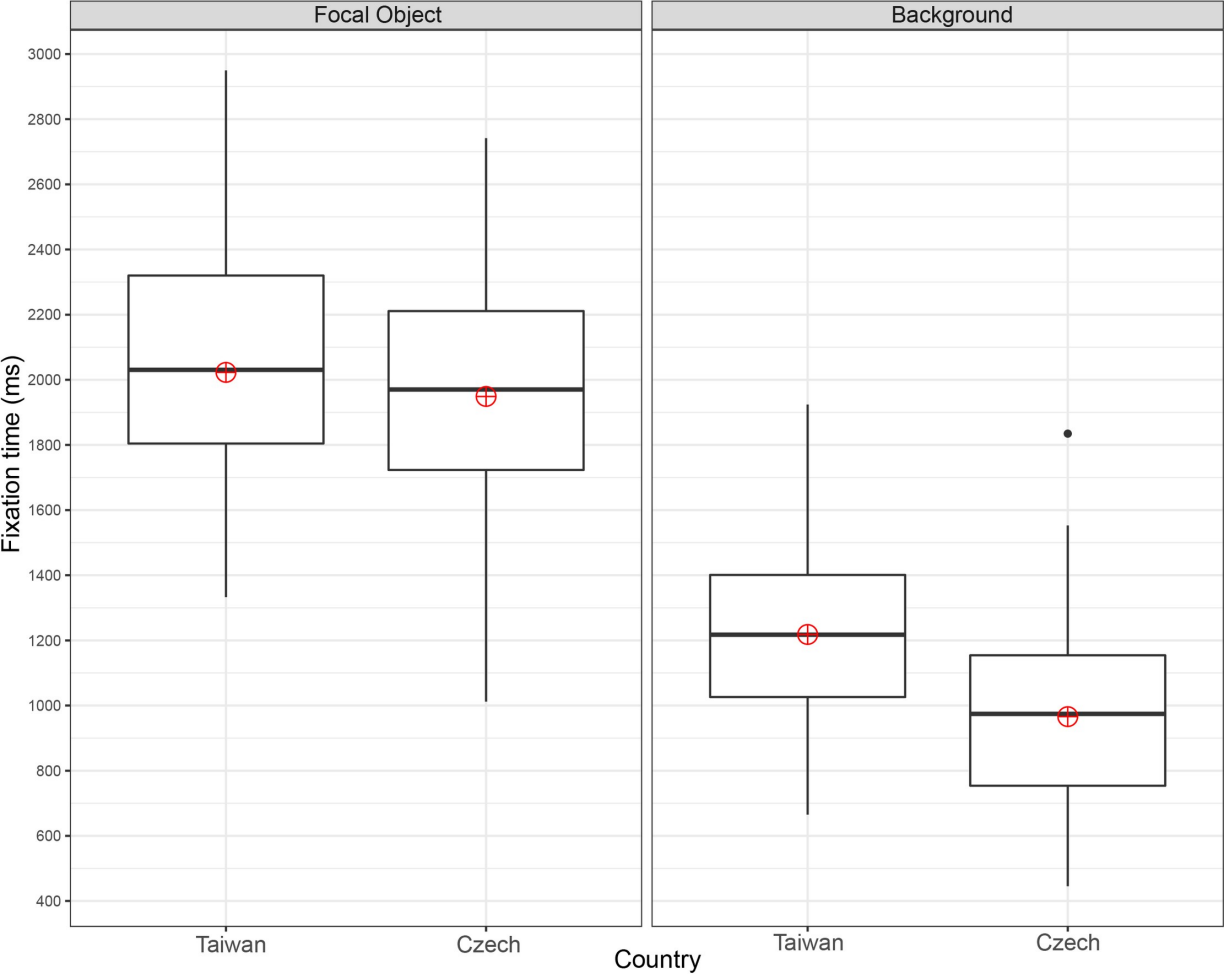
Mean fixation time: One focal object stimuli.

In fixation time, both groups spent more time observing the focal object than the background: F(1, 228) = 436.05, p = 2e-1616, ηp^2^ = 0.66. The main effect of culture was significant: F(1, 228) = 14.51, p = 0.00020002, ηp^2^ = 0.06., as was the interaction between culture and ROI type: F(1, 228) = 436.05, p = 0.038038, ηp^2^ = 0.02. No significant differences were found between the Czech (M = 1949, SD = 383) and Taiwanese (M = 2023, SD = 353) participants in focal object fixation time, t(114) = -1.080, p = 0.283, g = 0.20. The Taiwanese (M = 1218; SD = 290) fixated longer on the background than Czechs (M = 966, SD = 264) in background fixation time, t(114) = 4.903, p = 3.161e-0606, with a large effect size g = 0.90 (Fig 5). We also tested for focal object to background transitions. No differences in the number of transitions were found between the Czech (M = 3.7, SD = 0.7) and Taiwanese (M = 3.6, SD = 0.7) participants, t(114) = 0.494, p = 0.623, g = 0.09.

#### Two focal objects

The same analyses were performed for stimuli with two focal objects. The proportion of first fixations was not significantly different in the Taiwanese (M = 98.3, SD = 2.9) and Czech (M = 98.7, SD = 3.9) groups, t(114) = -0.628, p = 0.531, with negligible effect size g = -0.116. One focal object was significantly first fixated on by both cultural groups.

**Fig 6:**
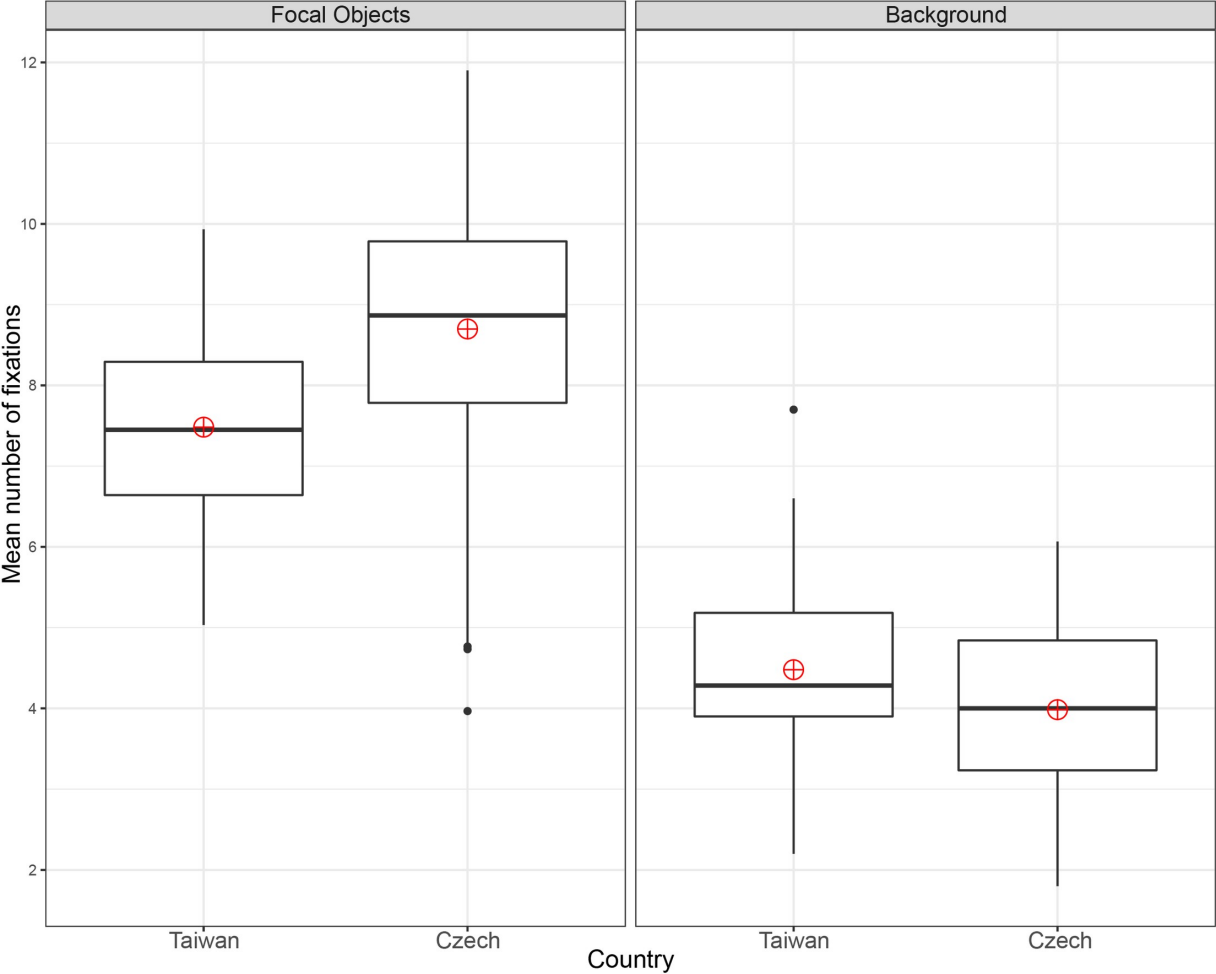
Mean number of fixations: Two focal objects stimuli.

In terms of the number of fixations, both groups fixated more on focal objects than backgrounds F(1, 228) = 532.04, p = 2e-1616, ηp^2^ = 0.70. The main effect of culture was significant: F(1, 228) = 4.65, p = 0.032, ηp^2^ = 0.02, as was the interaction between culture and ROI: F(1, 228) = 26.15, p = 6.7e-0707, ηp^2^ = 0.10. Fig 6 indicates that the Czechs (M = 8.7, SD = 1.7) made significantly more fixations on focal objects than the Taiwanese (M = 7.5, SD = 1.1), t(114) = 4.595, p = 1.123e-0505, g = 0.85. By contrast, the Taiwanese (M = 4.5, SD = 1.1) participants fixated on the background significantly more than their Czech counterparts (M = 4.0, SD = 1.1), t(114) = 2.417, p = 0.017, with small effect size g = 0.45.

**Fig 7:**
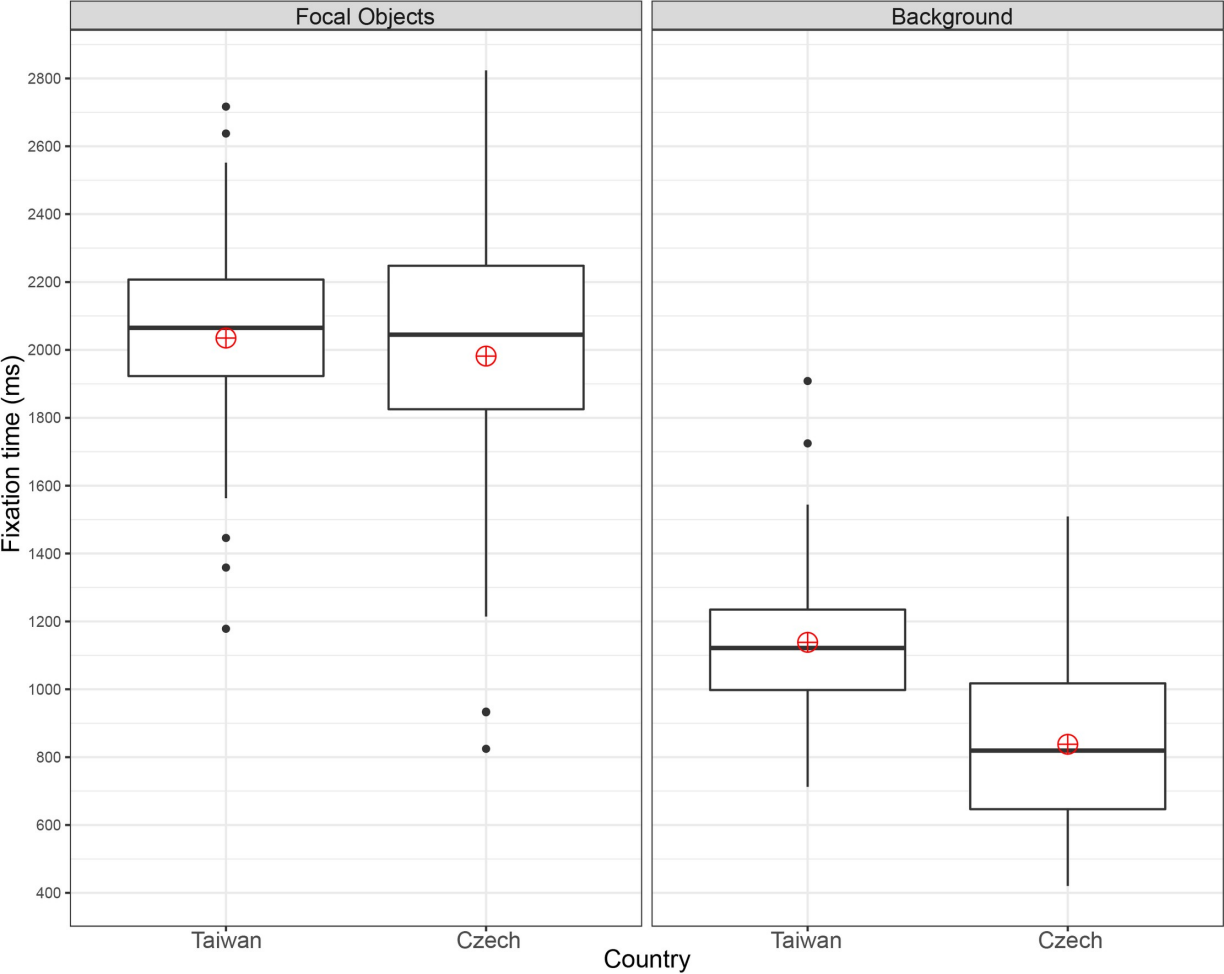
Mean fixation time: Two focal objects stimuli.

In terms of fixation time (Fig 7), both groups observed the focal objects longer than the background: F(1, 228) = 622.67, p = 2e-1616, ηp^2^ = 0.73. The main effect of culture was significant: F(1, 228) = 18.70, p = 2.29e-0505, ηp^2^ = 0.08., as was the interaction between culture and ROI type: F(1, 228) = 18.70, p = 0.002, ηp^2^ = 0.04. No significant differences were found between the Czech (M = 1982, SD = 436) and Taiwanese (M = 2035, SD = 299) participants in focal object fixation time, t(114) = -0.765, p = 0.446, g = 0.14. The Taiwanese participants (M = 1138; SD = 231) also fixated on backgrounds significantly longer than the Czechs (M = 838, SD = 235), t(114) = 6.953, p = 2.379e-1010, with large effect size g = 1.28.

Finally, the number of transitions in both groups was compared. In this case, both transitions between focal objects and background and transitions between focal objects were analysed. While no significant differences were consistently found in one focal object stimuli between the Czech (M = 3.7, SD = 0.8) and Taiwanese (M = 3.9, SD = 0.7) participants in the focal object to background transitions, t(114) = -1.322, p = 0.189, g = 0.24, the Czech participants (M = 2.4, SD = 0.8) transitioned significantly more between the two focal objects than the Taiwanese (M = 2.0, SD = 0.6), t(114) = 2.939, p = 0.004, g = 0.54.

### Analysis 3: Relationship between eye-movement metrics, CFT and other variables

Only data from respondents not excluded from one of the two previous analyses (CFT, complex scenes) were part of this analysis. We calculated 108 cases (56 Czech, 52 Taiwanese). To determine whether the eye-tracking metrics had any relationship with other collected variables, we calculated a relative focal object/background number of fixations, fixation times and number of saccades by dividing the focal object metrics of each participant with their background metrics. A series of three multiple linear regressions was subsequently performed on CFT global preference scores, sex, age, household size, experience with living abroad and nationality as independent variables, and relative number of fixations, relative fixation times and relative number of saccades (each in a separate model) as dependent variables. We expected a significant positive relationship between CFT global preference and the relative eye-tracking variables, meaning that the more global the attention patterns, the more the focal objects were focused on. We did not hypothesize any relationships between eye-tracking metrics and other variables included in the models for exploratory purposes.

Nationality was a significant predictor of the relative number of fixations: F(6, 101) = 2.82, Pr(>|t|) = 0.00239, with an adjusted R2 of 0.092, relative fixation time: F(6, 101) = 2.65, Pr(>|t|) = 0.00273, with an adjusted R2 of 0.085, and relative number of saccades: F(6, 101) = 2.25, Pr(>|t|) = 0.011, with an adjusted R2 of 0.065. No significant regression equation was found for any other variable in these three tested models. When nationality was excluded from the models, the adjusted R2 of the models had a maximum value of less than 0.006. That means the focal object to background eye-tracking patterns appeared to be independent of the global preference scores and sociodemographic variables mentioned above, except for the participants’ nationalities.

## Discussion

The present study introduces several new findings into the ongoing debate on cross-cultural similarities and differences in visual perception and cognition according to the theory of holistic and analytic cognitive styles [2, 29]. The study examined two dimensions of cognitive style: global vs. local attention (measured by CFT) and context sensitivity (measured by eye-tracking metrics as complex scenes were perceived). In summary, both Czech and Taiwanese showed a strong global preference effect in the CFT: global processing speed was significantly quicker compared to local feature processing. No significant differences were found between the groups in the global preference scores. No cultural differences were noted as affecting the proportion of first fixations on focal objects in the eye-tracking metrics calculated in the scene perception task. Both groups fixated more frequently on and spent more time observing the focal objects than the background. Czech participants fixated more frequently on focal objects than the Taiwanese, while the Taiwanese spent more time observing the background. The results were consistent across one and two focal object scenarios. No differences were found between the groups in the number of focal object/background transitions. The Czech participants made more direct transitions between focal objects in the dual focal object scenario. No significant relationships were found in the relative (focal object vs background) eye-tracking metrics, such as number of fixations, fixation time, number of saccades, CFT, global preference score, sex, age, household size or experience living abroad.

### Compound Figures Test

As mentioned above, global vs. local attention was investigated using a CFT [43]. The tasks consisted of two sub-tasks that would indicate a person’s tendency for global (attention to global characteristics) vs. local (attention to local characteristics) processing. From these two scores, a global preference score was calculated, which served as an indicator of global vs. local attention: the higher the score, the more globally oriented a person was. The results suggested that the Taiwanese in our sample perceived slightly more globally than the Czechs. The results, however, were not significant with small effect size, and therefore did not provide any strong evidence of cross-cultural differences such as those reported by McKone et al. [32]. The results were more in favour of the findings of a preregistered study conducted by Hakim et al. [24], which did not detect any cultural differences in global processing between American, international Asian and Chinese samples, or the study by von Mühlenen et al. [60], which did not detect any differences between samples from the UK and India.

It is important to note that the differences in results may have been caused by the methods of administration and precise nature of the task. Hakim et al. [24] instructed the participants to identify target letters (E or H) in compound letters while stimuli were presented centrally. The respondents only responded whether the target letters were present on the global or local levels, without reporting the specific target, and stimuli were displayed until a response was given. Von Mühlenen et al. [60] asked the participants to identify a specific target (H or T) by pressing the respective key. The Navon task was assigned in combination with emotionally charged (happy, neutral, sad) images to affect the emotional state of the participant. Stimuli were presented until a response was given or for a maximum of 5 seconds. McKone et al. [32] presented the stimuli laterally to test hemispherical differences in global/local processing. We therefore declare that it would be premature to draw final conclusions on the issue of cross-cultural differences in global vs. local attention.

An interesting effect observed in the global and local reaction times should be noted. Although both groups were slightly quicker in responding to global tasks than local tasks, the reaction times of the Taiwanese group were, in both cases, approximately twice as long. This finding replicated the results of the CFT reported by Lacko [45] in samples of Czech and continental Chinese participants. Chinese participants were also significantly slower. Since the test instructions in both Czech and traditional Chinese included the instruction to solve the task “as quickly as possible”, such a large difference in reaction times should not be an indicator of method bias [50, 61] but more probably of the differences in response styles between both samples. These differences in speed of response might also be explained by the avoidance of risk-taking behaviour inherent to Confucian ethics [62], or the notion of “losing face” typical for some Asian cultures [63]. These assumptions should be tested in future research, for example, by manipulating individual/group administration of the task, or with the administrator persona (e.g. Would the perceived social status of the administrator influence the tendency of the participants not to make mistakes?).

### Complex scenes

The second dimension in differences of perception, i.e. context sensitivity, was investigated by measuring eye-tracking patterns while complex real-world scenes were observed. Two types of real-world scenes were shown in which the number (one or two) of focal (perceptually salient) objects was changed. According to previous research [15, 36], we expected Czech participants to focus relatively more on focal objects (first fixations, number of fixations, fixation time) than the Taiwanese, and also, because of the expected holistic nature of eye-movement patterns of the Taiwanese, we assumed that they would make more transitions between various parts of the stimuli. Stimuli with one and two focal objects were analysed separately.

No significant cross-cultural differences were found in the percentage of first fixations on focal object(s). The results were consistent across both stimulus types. Both groups mostly first fixated on the focal object, which is consistent with previous findings [15, 23, 36] and suggests the prevalence of bottom-up perceptual processes soon after the stimulus is displayed. The early visual attention was mainly driven by the perceptual properties of the stimulus, and the subjects primarily fixated on highly salient objects [64].

In both the one and two focal object conditions, the Czech participants made significantly more fixations on the focal objects and spent less time fixating on the background than the Taiwanese. In the case of stimuli with two focal objects, the Taiwanese made significantly more background fixations. These results agree with the assumption that analytic perceivers focus more on the focal object and its properties and that holistic perceivers focus relatively more on the background [1]. The Czechs also transitioned significantly more between both focal objects, which might again be an indicator of relatively higher focus on objects [1]. However, contrary to our expectations, no cross-cultural differences in focal object to background transitions were detected in either the one or two focal object conditions. As holistic perceivers, if the Taiwanese observed the image as a “whole”, we would expect them to make more transitions.

The results showed that the Czechs made more transitions between focal objects. The main eye-tracking metrics (number of fixations, fixation time) in this study replicated the results of studies conducted by Chua et al. [36] and Duan et al. [15] and demonstrated the expected higher focus of Czechs on focal objects (number of fixations) and of Taiwanese on backgrounds (fixation time). As mentioned by Rayner et al. [26], it is questionable whether the number of the points of interest in the scene affected the scanning patterns across cultures. Our results showed that the cross-cultural differences in scanning patterns were consistent for both stimuli with one and two focal objects.

### Theoretical implications

The present study is one of few that have attempted to compare multiple components of cognitive styles within the framework of the general holistic–analytic model [1, 2]. It defines cognitive style as a bipolar dimension in which analytic perception is defined as rule-based, formally logical, field independent, with selective attention focused on salient objects and locally oriented, while the holistic pole is rather intuitive, dialectical, field dependent, sensitive to context, with attention focused on the “whole” and globally oriented. Two tasks were used to analyse cognitive style: hierarchical figures (global and local processing) and complex natural scenes (attention to object and background). Using linear regression, no significant relationships were found between the tasks, which is in line with other studies [8, 24] that used more methods to validate the analytic–holistic cognitive styles theory and found them unrelated.

The results raise questions of a) the validity of the concept of cognitive styles and b) its dichotomous nature. In terms of a), Cuneo et al. [65] tested the discriminant validity of methods for analytic–holistic style diagnostics and found that questionnaire methods overlapped with personality and that maximum performance methods (Group Embedded Figures Test) overlapped with intelligence. Further research should test the construct, concurrent and discriminant validity of cognitive styles, especially in relation to personality and intelligence. Some methods not based on maximum performance attempt to overcome this problem by using two independent tests: one for each of the opposing poles of cognitive styles. In terms of b), if the concept of cognitive styles is valid and non-overlapping with other constructs, its nature might be different from the possibly reductionist dichotomic analytic–holistic (or “East–West”) definition of the general holistic–analytic model. Kozhevnikov et al. [37] proposed an alternative hierarchical–ecological model of cognitive styles (see Introduction) that has the form of a hierarchical matrix in which cognitive style families are organized along levels of informational processing. According to this model, the different cognitive styles would not necessarily have to (cor)relate, because an environment might, for example, support the development of global processing (holistic characteristic) and focus on salient objects (analytic characteristic). This model might explain the lack of correlation between different methods of cognitive style analysis observed in this and some other studies [8, 24, 45]. Future research should therefore attempt to verify the hierarchical–ecological model [37] of cognitive styles and specify the number of cognitive style families. Conducting research on the stability/flexibility of cognitive styles and investigating the developmental aspects (e.g. children of different ages) of cognitive style and its adaptive nature (e.g. research on expatriates during the process of cultural adaptation) is also suggested.

### Limitations

This study carries some limitations. Most importantly, only student samples were used for this research. The potential differences in results obtained from student samples and the results from Czech and Taiwanese general population subgroups would be based on the adopted theoretical perspective. If we adopt the dichotomous approach of the general holistic–analytic model [1], which states that East is holistic and West is analytic, we would expect to find similar patterns in the similarities and differences between other Czech and Taiwanese subpopulations. We might also expect larger effect sizes if more diverse subpopulations (e.g. uneducated individuals, children, seniors) are compared. However, if we adopt the approach of the hierarchical–ecological model [37], we could expect substantially different patterns of global attention or context sensitivity in different subgroups, because these subgroups might mature and live in fundamentally different social and physical environments that require distinct ways of cognitive adaptation. Furthermore, a study by Waxman et al. [66] showed evidence of cross-culturally divergent developmental changes in attentional patterns. Our results, therefore, are not generally applicable to all citizens of the Czech Republic or Taiwan.

Potential differences in eye-tracking systems in the Czech Republic and Taiwan need to be mentioned among the limitations of our study. However, we gave special attention to this issue throughout all stages of the research to eliminate any possible confounding effects and assure full equivalence in measurement. Both eye-tracking systems were set to the same sampling frequency. Both eye-tracking systems also had similar spatial accuracy and precision [67]. The spatial accuracy threshold was the same for both measurements (max. 1° of visual angle), and the calibration error was the same in both samples (0.56° of visual angle). Fixation calculations were also conducted simultaneously for both datasets in Ogama software, and the ROIs were the same for both cultural groups. Only robust eye-tracking metrics (number of fixations, fixation time, transitions in ROIs) were calculated [49], and the ROI specification of the scenes was binary in character (figure vs. background). Therefore, the size of the stimuli (figures) used in this experiment was two levels higher (approx. 10° of visual angle) than the variability in accuracy of eye-tracking in the participants (approx. 0.1° of visual angle). Therefore, the interference in data caused by using two eye-tracking systems can be considered negligible in our research design.

Another point to consider is the nature of the task, as it might be a method factor that affects eye-movement patterns and potential cross-cultural differences in these patterns. As previously mentioned, the eye-movement task was an implicit, free-viewing task for evaluating the “aesthetic preferences” of each image. It should be noted that the eye-movement patterns might differ depending on the nature of the task, as demonstrated by Yarbus [68] in his seminal monograph. In his qualitative study, instructions were manipulated and the differences in eye-movement patterns were subsequently observed. Castelhano et al. [69] found differences in aggregate eye-movement metrics depending on whether the observers searched for a target or memorized the stimuli. The need to consider the nature of the task while evaluating cross-cultural differences is also emphasized by Alotaibi et al. [16]. Greene et al. [70], however, demonstrated quantitatively with pattern classifiers that the task-related effects on scene viewing might be overrated, at least in the case of brief presentations of stimuli, when an observer’s gaze seems to be mostly driven by the saliency of various parts of the scene. Nevertheless, the observed similarities and differences between both groups in eye-movements should not be generalized to other possible scene perception designs (e.g. passive viewing, visual search or recognition).

While some studies reported differences between Americans and Chinese [36] and Chinese and Africans in a free-viewing task [15], studies conducted by Rayner et al. [25] combined with a memory task showed negative results. Similarly, no cross-cultural differences were found in a change-blindness experiment performed by Masuda et al. [34]. Future eye-tracking research exploring the perception of complex scenes should attempt to combine several tasks (e.g. free-viewing, visual searches, flicker-tasks).

## References

[1] Nisbett R, Masuda T. Culture and point of view. Proc Natl Acad Sci U S A. 2003;100(19): 11163–11170. 10.1073/pnas.1934527100 12960375 PMC196945

[2] Nisbett R, Peng K, Choi I, Norenzayan A. Culture and systems of thought: holistic versus analytic cognition. Psychol Rev. 2001;108(2): 291–310. 10.1037/0033-295x.108.2.291 11381831

[3] Bornstein MH, Toda S, Azuma H, Tamis-LeMonda C, Ogino M. Mother and infant activity and interaction in Japan and in the United States: II. A comparative microanalysis of naturalistic exchanges focused on the organization of infant attention. Int J Behav Dev. 1990;13(3): 289–308.

[4] Han JJ, Leichtman MD, Wang Q. Autobiographical memory in Korean, Chinese, and American children. Dev Psychol. 1998;34(4): 701–713. 10.1037//0012-1649.34.4.701 9681262

[5] Grossmann I, Varnum M. Social class, culture, and cognition. Soc Psychol Personal Sci. 2010;000(00): 1–9.

[6] Ventura P, Pattamadilok C, Fernandes T, Klein O, Morais J, Kolinsky R. Schooling in western culture promotes context-free processing. J Exp Child Psychol. 2008;100(2): 79–88. 10.1016/j.jecp.2008.02.001 18343399

[7] Miyamoto Y, Nisbett R, Masuda T. Culture and the physical environment holistic versus analytic perceptual affordances. Psychol Sci. 2006;17(2): 113–119. 10.1111/j.1467-9280.2006.01673.x 16466418

[8] Kӧster M, Castel J, Gruber T, Kärtner J. Visual cortical networks align with behavioral measures of context-sensitivity in early childhood. NeuroImage, 2017;163: 413–418. 10.1016/j.neuroimage.2017.08.008 28780400

[9] Tang Y, Zhao L, Lou Y, Shi Y, Fang R, Lin X, et al Brain structure differences between Chinese and Caucasian cohorts: A comprehensive morphometry study. Hum Brain Mapp. 2018;39(5): 2147–2155. 10.1002/hbm.23994 29400417 PMC6625506

[10] Uskul A, Kitayama S, Nisbett R. Ecocultural basis of cognition: Farmers and fishermen are more holistic than herders. Proc Natl Acad Sci U S A. 2008;105(25): 8552–8556. 10.1073/pnas.0803874105 18552175 PMC2438425

[11] Dong X, Talhelm T, Ren X. Teens in rice county are more interdependent and think more holistically than nearby wheat county. Soc Psychol Personal Sci. 2019;10(7): 966–976.

[12] Ishii K. Culture and the mode of thought: A review. Asian J Soc Psychol. 2013;16(2): 123–132.

[13] Ji L-J, Schwarz N, Nisbett R. Culture, autobiographical memory, and behavioral frequency reports: Measurement issues in cross-cultural studies. Pers Soc Psychol Bull. 2000;26(5): 585–593.

[14] Oyserman D, Lee S. Does culture influence what and how we think? Effects of priming individualism and collectivism. Psychol Bull. 2008;134(2): 311–342. 10.1037/0033-2909.134.2.311 18298274

[15] Duan Z, Wang F, Hong J. Culture shapes how we look: Comparison between Chinese and African university students. J Eye Mov Res. 2016;9(6): 1–10.

[16] Alotaibi A, Underwood G, Smith AD. Cultural differences in attention: Eye movement evidence from a comparative visual search task. Conscious Cogn. 2017;55: 254–265. 10.1016/j.concog.2017.09.002 28946046

[17] Arieli S, Sagiv L. Culture and problem-solving: Congruency between the cultural mindset of individualism versus collectivism and problem type. J Exp Psychol Gen. 2018;147(6): 789–814. 10.1037/xge0000444 29888937

[18] Kardan O, Shneidman L, Krogh-Jespersen S, Gaskins S, Berman M, Woodward A. Cultural and developmental influences on overt visual attention to videos. Sci Rep. 2017;7(1): 11264 10.1038/s41598-017-11570-w 28900172 PMC5595807

[19] Knight K, Nisbett R. Culture, class, and cognition: Evidence from Italy. J Cogn Cult. 2007;7(3): 283–291.

[20] Tutnjevič S, Lakič S. Language-mediated object categorization: A longitudinal study with 16-to 20-month-old Serbian-speaking children. Eur J Dev Psychol. 2018;15(5): 608–622.

[21] Stachoň Z, Šašinka Č, Čeněk J, Štěrba Z, Angsuesser S, Fabrikant S, et al Cross-cultural differences in figure-ground perception of cartographic stimuli. Cartogr Geogr Inf Sci. 2019;46(1): 82–94.

[22] Kitayama S, Ishii K, Imada T, Takemura K, Ramaswamy J. Voluntary settlement and the spirit of independence: Evidence from Japan's “northern frontier”. J Pers Soc Psychol. 2006; 91(3), 369–384. 10.1037/0022-3514.91.3.369 16938025

[23] Evans K, Rotello C, Li X, Rayner K. Short Article: Scene perception and memory revealed by eye movements and receiver-operating characteristic analyses: Does a cultural difference truly exist? Q J Exp Psychol. 2009;62(2): 276–285. 10.1080/17470210802373720 PMC2668147 18785074

[24] Hakim N, Simons D, Zhao H, Wan X. Do easterners and westerners differ in visual cognition? A preregistered examination of three visual cognition tasks. Soc Psychol Personal Sci. 2017;8(2): 142–152.

[25] Rayner K, Castelhano M, Yang J. Eye movements when looking at unusual/weird scenes: Are there cultural differences? J Exp Psychol. 2009;35(1): 254–259. 10.1037/a0013508 19210095 PMC2668126

[26] Rayner K, Li X, Williams C, Cave K, Well A. Eye movements during information processing tasks: Individual differences and cultural effects. Vision Res. 2007;47(21): 2714–2726. 10.1016/j.visres.2007.05.007 17614113 PMC2048814

[27] Stachoň Z, Šašinka Č, Čeněk J, Angsüsser S, Kubíček P, Štěrba Z, et al Effect of Size, Shape and Map Background in Cartographic Visualization: Experimental Study on Czech and Chinese Populations. ISPRS Int J Geoinf. 2018;7(11): 1–15.

[28] Chiu LH. A cross-cultural comparison of cognitive styles in Chinese and American children. Int J Psychol. 1972;7(4): 235–242.

[29] Norenzayan A, Smith E, Kim B, Nisbett R. Cultural preferences for formal versus intuitive reasoning. Cogn Sci. 2002;26(5): 653–684.

[30] Peng K., Nisbett R. Culture, dialectics, and reasoning about contradiction. Am Psychol. 1999;54(9): 741–754.

[31] Kühnen U, Hannover B, Roeder U, Shah AA, Schubert B, Upmeyer A, et al Cross-cultural variations in identifying embedded figures: Comparisons from the United States, Germany, Russia and Malaysia. J Cross Cult Psychol. 2001;32(3):365–371.

[32] McKone E, Aimola DA, Fernando D, Aalders R, Leung H, Wickramariyaratne T, et al Asia has the global advantage: Race and visual attention. Vision Res. 2010;50(16): 1540–1549. 10.1016/j.visres.2010.05.010 20488198

[33] Masuda T, Nisbett R. Culture and change blindness. Cogn Sci. 2006;30(2): 381–399. 10.1207/s15516709cog0000_63 21702819

[34] Masuda T, Ishii K, Kimura J. When does the culturally dominant mode of attention appear or disappear? Comparing patterns of eye movement during the visual flicker task between European Canadians and Japanese. J Cross Cult Psychol. 2016;47(7): 997–1014.

[35] Steinmetz KR, Sturkie CM, Rochester NM, Liu X, Gutchess AH. Cross-cultural differences in item and background memory: examining the influence of emotional intensity and scene congruency. Memory. 2017;26(6): 751–758. 10.1080/09658211.2017.1406119 29173027

[36] Chua H, Boland J, Nisbett R. Cultural variation in eye movements during scene perception. Proc Natl Acad Sci U S A. 2005;102(35): 12629–12633. 10.1073/pnas.0506162102 16116075 PMC1194960

[37] Kozhevnikov M, Evans C, Kosslyn SM. Cognitive style as environmentally sensitive individual differences in cognition: A modern synthesis and applications in education, business, and management. Psychol Sci Public Interest. 2014;15(1): 3–33. 10.1177/1529100614525555 26171827

[38] Nosal CS. Psychologiczne modele umysłu [Psychological models of the mind]. Warsaw: PWN; 1990.

[39] Hofstede G. Culture's consequences: Comparing values, behaviors, institutions and organizations across nations London: Sage publications; 2001.

[40] Caparos S, Linnell K, Bremner A, de Fockert J, Davidoff J. Do local and global perceptual biases tell us anything about local and global selective attention? Psychol Sci. 2013;24(2): 206–212. 10.1177/0956797612452569 23300230

[41] Kiyokawa S, Dienes Z, Tanaka D, Yamada A, Crowe L. Cross cultural differences in unconscious knowledge. Cognition. 2012;124(1): 16–24. 10.1016/j.cognition.2012.03.009 22560768

[42] Nisbett R, Miyamoto Y. The influence of culture: holistic versus analytic perception. Trends Cogn Sci. 2005;9(10): 467–473. 10.1016/j.tics.2005.08.004 16129648

[43] Navon D. Forest before trees: The precedence of global features in visual perception. Cogn Psychol. 1977;9(3): 353–383.

[44] Kukaňová M. Porovnání dvou typů vizualizací z hlediska percepční a kognitivní zátěže a kognitivních schopností jedince. [Comparison of the two types of visualization in terms of perceptual and cognitive load, and personal cognitive abilities]. Doctoral Dissertation. Brno: Masaryk University. 2017. Available online: https://is.muni.cz/auth/th/djk1o/

[45] Lacko D. Individuální a interkulturní rozdíly ve vnímání a myšlení. [The individual and intercultural differences in perception and cognition]. Master Thesis. Brno: Masaryk University. 2018. Available online: https://is.muni.cz/th/cf1aq/

[46] Opach T, Popelka S, Dolezalova J, Rød JK. Star and polyline glyphs in a grid plot and on a map display: which perform better? Cartogr Geogr Inf Sci. 2018;45(5): 400–419.

[47] Šašinka Č, Stachoň Z, Kubíček P, Tamm S, Matas A, Kukaňová M. The Impact of Global/Local Bias on Task-Solving in Map-Related Tasks Employing Extrinsic and Intrinsic Visualization of Risk Uncertainty Maps, Cartogr J. 2019; 1–17.

[48] Šašinka Č, Morong K, Stachoň Z. The Hypothesis platform: An online tool for experimental research into work with maps and behavior in electronic environments. ISPRS Int J Geoinf. 2017;6(12): 1–22.

[49] Popelka S, Stachoň Z, Šašinka Č, Doležalová J. EyeTribe tracker data accuracy evaluation and its interconnection with Hypothesis software for cartographic purposes. Comput Intell Neurosci. 2016: 1–14. 10.1155/2016/9172506 27087805 PMC4819090

[50] Čeněk J, Urbánek T. Adaptace a ekvivalence testových metod: Inspirace pro psychologické testování minorit v ČR. [The adaptation and equivalence of test methods: Inspiration for psychological assessment of minorities in the Czech Republic]. Czechoslovak Psychol. 2019;63(1): 42–54.

[51] Ægisdóttir S, Gerstein LH, Çinarbaş DC. Methodological issues in cross-cultural counselling research: Equivalence, bias, and translations. Couns Psychol. 2008;36(2): 188–219.

[52] Van de Vijver F, Tanzer NK. Bias and equivalence in cross-cultural assessment. Eur Rev Appl Psychol. 1998;47(4): 263–279.

[53] Faul F, Erdfelder E, Lang A-G, Buchner A. G*Power 3: A flexible statistical power analysis program for the social, behavioral, and biomedical sciences. Behav Res Methods. 2007;39: 175–191. 10.3758/bf03193146 17695343

[54] R Core Team. R: A language and environment for statistical computing. R Foundation for Statistical Computing, Vienna (Austria); 2017 URL https://www.R-project.org/.

[55] Voßkühler A, Nordmeier V, Kuchinke L, Jacobs AM. OGAMA (Open Gaze and Mouse Analyzer): Open-source software designed to analyze eye and mouse movements in slideshow study designs. Behav Res Methods. 2008;40(4): 1150–1162. 10.3758/BRM.40.4.1150 19001407

[56] Caparos S, Fortier-St-Pierre S, Gosselin J, Blanchette I, Brisson B. The tree to the left, the forest to the right: Political attitude and perceptual bias. Cognition. 2015;134: 155–164. 10.1016/j.cognition.2014.10.006 25460388

[57] de Fockert JW, Cooper A. Higher levels of depression are associated with reduced global bias in visual processing. Cogn Emot. 2014;28(3): 541–549. 10.1080/02699931.2013.839939 24067089

[58] Salvucci DD, Goldberg JH. Identifying fixations and saccades in eye-tracking protocols In Duchowski AT, editor. Proceedings of the Eye Tracking Research and Applications Symposium. New York: ACM Press; 2000 pp. 71–78.

[59] Popelka S. Eye-tracking (nejen) v kognitivní kartografii: Praktický průvodce tvorbou a vyhodnocením experimentu [Eye-tracking (not only) in cognitive cartography: A practical guide for creation and analysis of an experiment]. Olomouc (CZ): UPOL; 2018.

[60] von Mühlenen A, Bellaera L, Singh A, Srinivasan N. The effect of sadness on global-local processing. Atten Percept Psychophys. 2018;80(5): 1072–1082. 10.3758/s13414-018-1534-7 29729000

[61] He J, Van de Vijver F. A general response style factor: Evidence from a multi-ethnic study in the Netherlands. Pers Individ Dif. 2013;55(7): 794–800.

[62] Hong LK. Risky shift and cautions shift: Some direct evidence on culture-value theory. Soc Psychol. 1978;41(4): 342–346.

[63] Dong Q, Lee Y-FL. The Chinese concept of face: A perspective for business communicators. Bus Soc. 2007;20(1/2): 204–216.

[64] Spotorno S, Faure S. Change detection in complex scenes: Hemispheric contribution and the role of perceptual and semantic factors. Perception. 2011;40(1): 5–22. 10.1068/p6524 21513180

[65] Cuneo F, Antonietti JP, Mohr C. Unkept promises of cognitive styles: A new look at old measurements. PLoS One. 2018;13(8):e0203115 10.1371/journal.pone.0203115 30153302 PMC6112650

[66] Waxman SR, Fu X, Ferguson B, Geraghty K, Leddon E, Liang J, et al How early is infants' attention to objects and actions shaped by culture? New evidence from 24-month-olds raised in the US and China. Front Psychol. 2016;7(97): 1–10. 10.3389/fpsyg.2016.00097 26903905 PMC4742528

[67] Orquin JL, Holmqvist K. Threats to the validity of eye-movement research in psychology. Behav Res Methods. 2018;50: 1645–1656. 10.3758/s13428-017-0998-z 29218588

[68] Yarbus AL. Eye movements and vision New York: Springer; 1967.

[69] Castelhano MS, Mack ML, Henderson JM. Viewing task influences eye movement control during active scene perception. J Vis. 2009;9(3): 1–15. 10.1167/9.3.6 19757945

[70] Greene MR, Liu T, Wolfe JM. Reconsidering Yarbus: A failure to predict observers’ task from eye movement patterns. Vision Res. 2012;62: 1–8. 10.1016/j.visres.2012.03.019 22487718 PMC3526937

