# Correction: Cultural variations in global and local attention and eye-movement patterns during the perception of complex visual scenes: Comparison of Czech and Taiwanese university students

**DOI:** 10.1371/journal.pone.0247219

**Published:** 2021-02-11

**Authors:** Jiří Čeněk, Jie-Li Tsai, Čeněk Šašinka

The ORCID iD is missing for the second author. Author Jie-Li Tsai’s ORCID iD is: 0000-0001-5413-4889
